# Successful Treatment of Severe Hepatopulmonary Syndrome as a Rare Complication of Zellweger Spectrum Disorder

**DOI:** 10.1002/jmd2.70026

**Published:** 2025-08-28

**Authors:** Riya Mary Tharakan, Sanjay Rajwal, Bernd C. Schwahn

**Affiliations:** ^1^ Manchester Centre for Genomic Medicine, St Mary's Hospital Manchester University NHS Foundation Trust, Health Innovation Manchester Manchester UK; ^2^ Paediatric Liver Unit Leeds Children's Hospital Leeds UK; ^3^ Division of Evolution & Genomic Sciences, School of Biological Sciences, Faculty of Biology, Medicine and Health University of Manchester Manchester UK

**Keywords:** hepatopulmonary syndrome, liver transplantation, peroxisomal biogenesis disorder, Zellweger spectrum disorder

## Abstract

We report the case of an 11‐year‐old girl who developed hepatopulmonary syndrome (HPS) as a rare complication of Zellweger spectrum disorder and was successfully treated with liver transplantation. Our patient presented with neonatal sensorineural hearing loss. Muscular hypotonia, global developmental delay, and pigmentary retinopathy in infancy led to a diagnosis of peroxisomal biogenesis disorder due to compound heterozygous *PEX1* variants. Despite feeding disorder, poor weight gain, mild liver disease with subclinical coagulopathy, she had a relatively uneventful course, attaining developmental milestones till 7 years of age, when she was noted to have persistent central cyanosis (TcSO_2_ 72%) with poor oxygen response. Echocardiogram and CT chest were normal. Liver ultrasound demonstrated mild portal hypertension with a small spleen. An ultrasound bubble test established extracardiac right‐left shunting, and perfusion scintigraphy confirmed the diagnosis of HPS. Angiography showed increased portal pressure with normal right atrial pressures, ruling out porto‐pulmonary hypertension. Due to the limited prognosis of HPS and inadequate oxygenation on 2 L/min oxygen supplementation, after multidisciplinary discussion, a decision was made to proceed with an orthotopic liver transplant (OLT). Seven months later, she underwent OLT, following which her saturation normalized. At age 11 years, she continues to be clinically stable without oxygen supplementation. HPS being a rare complication of liver disease, is not easily recognized in the pediatric population. OLT proved beneficial in this child with an intermediately severe disorder of peroxisomal biogenesis.

**Summary**

- Hepatopulmonary syndrome is a rare, life‐threatening complication of chronic liver disease and can be successfully treated by orthotopic liver transplantation, even in a child with a multisystemic disorder of peroxisomal biogenesis.

## 1 Introduction

Zellweger spectrum disorder (ZSD) is the most common clinical presentation of the ultrarare peroxisomal disorders in infancy, with a prevalence of 1 in 50 000 live births [1]. Peroxisome assembly proteins called peroxins, encoded by *PEX* genes, are crucial for the formation of peroxisomes. Sequence variants in at least 13 different *PEX* genes have been associated with ZSD, but variants in *PEX1* and *PEX6* account for approximately 65% of patients [2]. Depending on the severity of the gene variant and resultant amount of residual protein action, the symptoms of affected children with ZSD vary significantly. Historically, infantile presentations were assigned to one of three clinical groups—Zellweger syndrome, Neonatal adrenoleukodystrophy, and infantile Refsum disease. However, recent subject reviews suggest the terms severe, intermediate, and mild ZSD [3].

Common manifestations of ZSD include severe developmental delay, neurological impairment including seizures, renal cysts, hepatic dysfunction, GI bleeding, adrenal insufficiency, and recurrent infections. Children presenting in the neonatal period have a very poor prognosis and usually die within the first year of life. Patients who present in later childhood have better chances of survival but can develop progressive liver disease and subsequent liver failure [4]. The severity of liver involvement varies from hepatomegaly and transaminitis to fibrosis, cirrhosis, and hepatocellular carcinoma [5].

Hepatopulmonary syndrome (HPS) was first described in 1977 as a rare complication of liver cirrhosis and portal hypertension [6]. The precise mechanism of HPS development is not fully understood, but there is evidence that portal hypertension is the main driving force. It has been suggested that splanchnic vascular overload due to hyperdynamic circulation leads to the release of endotoxins and cytokines, causing overproduction of vasodilatory substances such as nitric oxide, which promotes dilatation of pulmonary capillaries, resulting in a ventilation–perfusion mismatch [7]. Clinical symptoms include progressive dyspnea and cyanosis, with poor response to oxygen supplementation. HPS has not been previously reported in patients with peroxisomal disorders.

The currently accepted diagnostic criteria for HPS [8] are: (1) Presence of portal hypertension or liver failure; (2) decrease of arterial PO_2_ (PaO_2_ < 70 mmHg), or increased age‐corrected alveolar–arterial oxygen gradient on room air; and (3) presence of intrapulmonary vascular dilatations (IPVD) producing an intrapulmonary shunt. HPS prevalence in children with liver disease has generally been reported to be about 4%–20% [9, 10, 11]. However, in a study on children with cirrhosis in Brazil, HPS was found to be as high as 42.5% [12].

## 2 Case Report

### 2.1 Diagnosis and Management

Our patient presented to the hospital in her neonatal period due to a failed hearing screen, later confirmed to be sensorineural hearing loss. On further evaluation, she was also found to have muscular hypotonia, global developmental delay, and pigmentary retinopathy with moderate visual impairment. Magnetic resonance imaging showed suspected white matter changes.

The combination of symptoms suggested a peroxisomal disorder and she was referred to the metabolic service at the age of 19 months. Laboratory testing showed a normal liver profile (ALT 29 U/L Reference Interval (RI) 5–40) and mild coagulopathy (PT 18.4s RI 13.0–15.3, APTT 38.4s RI 28.6–35.8) and increased very long chain fatty acids and phytanic acid (Table 1). Genetic testing revealed heterozygosity for two previously reported pathogenic *PEX1* variants: c.2097dupT and c.2528G > A p.(Gly843Asp). The latter has been found in 25%–40% of ZSS patients and is associated with a mild phenotype [13, 14].

**TABLE 1:** Peroxisomal biomarkers before and after liver transplantation. Liver transplantation was performed at the age 7 years 10 months.

Analyte	C24/C22	C26/C22	C26	Phytanate	Pristanate	ALT	gGT	Bilirubin	Comments
Ref interval units	0.69–1.00 ratio	0.007–0.028 ratio	0.43–1.27 μmol/L	0–16 μmol/L	0–5 μmol/L	5–40 U/L	6–42 U/L	0–21 μmol/L	
Age									
1 year 7 months	1.23	0.283	14.26	101.0	15.5	33		< 3	Regular diet
2 years 3 months	—	—	—	160.9	39.6	36		5	
3 years 10 months	1.74	0.359	8.29	96.0	23.0	52	38	13	Low phytanate diet
4 years 8 months	—	—	—	113.8	30.8	52		14	
6 years 4 months	—	—	—	—	—	70	80	14	On formula feeds
7 years 2 months	1.59	0.298	13.34	16.4	1.9	57	55	30	
7 years 9 months	—	—	—	—	—	46	42	29	Liver transplantation
8 years 10 months	—	—	—	—	—	47	43	5	
10 years 2 months	—	—	—	—	—	29	20	4	
11 years 4 months	1.28	0.090	4.95	1.4	0.1	17	21	4	On formula and blended diet

*Note:* Concentrations of very long chain fatty acids, phytanate, and pristanate in plasma are given in μmol/L with the respective reference intervals in brackets.

The patient received a cochlear implant by the age of 22 months, and symptomatic management for ZSD was initiated at the age of 23 months, including supplementation with Vitamin K, ursodeoxycholic acid, chenodeoxycholic acid, and dietary phytanate restriction with an essential fatty acid supplement. Her coagulopathy showed some improvement but no normalization over subsequent years. The ALT was increased from the age of 3.5 years and remained in a range of 45–108 U/L with an upward trend, whereas bilirubin initially remained in the normal range. Hydrocortisone was substituted in physiological doses from the age of 4 years following a failed Synacthen test.

She developed recurrent nose bleeds at 4 years of age with the appearance of telangiectasia in the nasal cavity, which eventually settled within a year. Imaging to rule out systemic arteriovenous malformations, including abdominal ultrasound and a whole‐body contrast CT scan, showed normal results. Her psychomotor development progressed slowly. Gradually, she was able to walk with a frame, she could understand words, and was able to communicate with signs. She continued to have feeding difficulty for which oral formula supplements were tried. The low phytanate diet was relaxed due to poor food intake and poor weight gain at this time. Following further significant weight loss over the next 2 years, supplementary nasogastric feeding was initiated, and a gastrostomy was created at the age of 6 years. The supplementation with chenodeoxycholic acid was discontinued by 7 years of age due to changes in policy. She sustained pathological fractures at the age of 6 years 4 months and 7 years 9 months.

### 2.2 Onset and Diagnosis of Hepatopulmonary Syndrome

**FIGURE 1:**
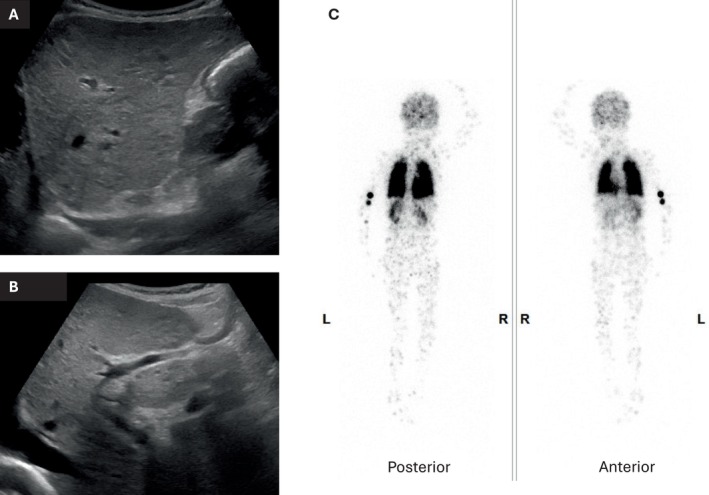
Imaging results at the age of 7 years 3 months. (A) Abdominal ultrasound scan showing nodular liver surface. (B) Abdominal ultrasound scan demonstrating recanalization of the ductus venosus. (C) 99 m‐Tc MAA scan demonstrating uptake of tracer in both kidneys and the brain, with a calculated right‐to‐left shunt of 39.8%.

At the age of 7 years 2 months, recurrent cyanosis of the fingertips and lips as well as intermittently raised temperatures were noted. She was admitted with suspected upper airway infection. On evaluation, she appeared to be well but dyspnoeic and hypoxemic with a tcSO_2_ of 70%. She was noted to be hyperventilating with low pCO_2_ and poor response to oxygen supplementation. Infection markers, viral screens, and chest X‐ray were unremarkable. Echocardiograms performed on day 6 and day 9 of admission showed no significant pulmonary hypertension but rapid opacification of the left heart after the fourth beat following IV injection of shaken saline solution in the absence of an intracardiac shunt, thereby suggesting the presence of an extracardiac right‐to‐left shunt. A subsequent chest CT angiogram excluded thoracic arteriovenous malformation. The liver ultrasound demonstrated a nodular liver surface (Figure 1A) with no relevant portosystemic or intrahepatic shunt. It did, however, reveal recanalization of the ductus venosus and a prominence of the superior mesenteric vein (Figure 1B) as indirect signs of persistent portal hypertension, although with a small spleen.

The possibility of HPS was considered and a macro‐aggregated albumin lung perfusion scan (99m‐Tc MAA) demonstrated significant accumulation of the tracer in the brain and kidneys with a calculated right to left shunt of 39.8%, confirming the diagnosis (Figure 1C).

### 2.3 Liver Transplantation and Outcome

The patient was transferred to the pediatric liver transplant unit, approximately a month after admission. Repeat USS demonstrated a nodular structure of the liver. A needle biopsy was performed, alongside an invasive angiography which demonstrated a free hepatic venous pressure of 7 mmHg and an increase to 22 mmHg after wedging. An attempt to treat with pentoxifylline was not made. The patient remained oxygen dependent and was maintained at oxygen saturation levels of 85%, with intermittent desaturations, with supplementation of 2 L/min of oxygen via nasal cannula.

**FIGURE 2:**
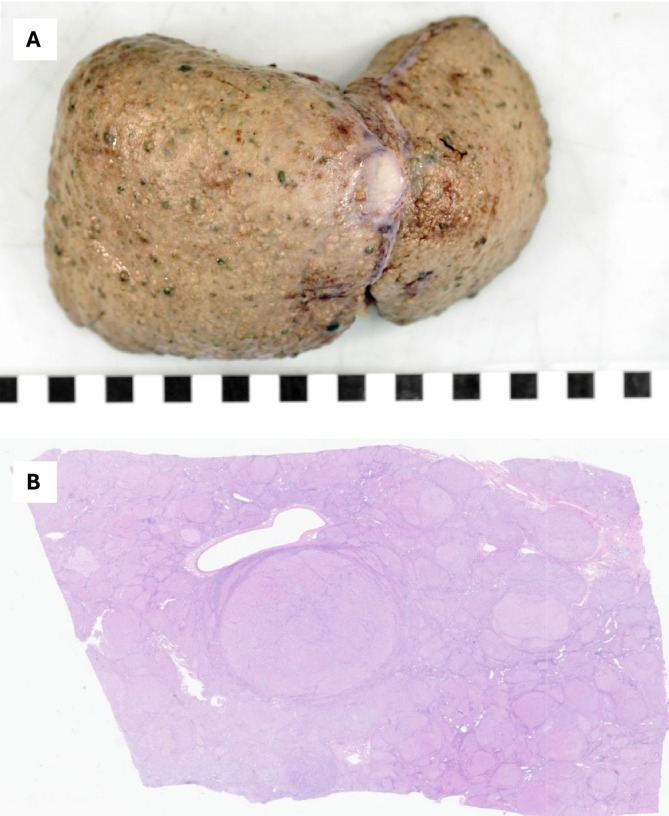
Macroscopic (A) and microscopic (B) view of the explanted liver, showing evidence of nodular cirrhosis.

After multidisciplinary discussion and with parental consent, the decision was made to proceed with liver transplantation despite a high risk of perioperative mortality and general compromise from the peroxisomal multisystem disease. Seven months later, at the age of 7 years 10 months, the patient underwent an orthotopic living‐related liver transplant. The explanted liver showed macroscopic and microscopic features of liver cirrhosis (Figure 2). After an uncomplicated postoperative course, the oxygen saturation slowly increased and completely normalized over the course of 2 months.

Now, almost 4 years later, she continues to remain clinically well without an oxygen requirement. Her liver function is normal. Biomarkers improved after transplantation (Table 1). Despite improved metabolic markers after liver transplantation, we observed a gradual progression of the retinal dystrophy with increasing visual impairment and development of a variable degree of resting tremor in both hands.

## 3 Discussion

HPS is a typical complication of chronic liver disease but can be difficult to diagnose at an early stage before progressive dyspnoea sets in. In a young child with a multisystemic metabolic disease, respiratory symptoms may rather be ascribed to infection, primary lung pathology, or secondary to cardiac pathology. However, making the right diagnosis is crucial in providing appropriate treatment.

The symptoms and signs can be mistaken for porto‐pulmonary hypertension (POPH), which is also a known complication of liver cirrhosis and portal hypertension. However, the pathophysiology here is the progressive remodeling of the small pulmonary arteries, along with vasoconstriction and thickening of the arterial wall which causes pulmonary arterial hypertension (PAH) and right heart failure [15]. The key difference between the two is the presence of intrapulmonary vasodilatation (IPVD) and the absence of PAH and right heart failure in HPS.

Demonstration of IPVD is most effectively done by transthoracic echocardiography that is contrast‐enhanced by injecting microbubbles from agitated saline. Alternatively, radionucleotide lung perfusion scanning, using technetium labeled macroaggregated albumin particles, can also be used [16]. To screen for the presence of HPS, pulse oximetry has been proposed as a non‐invasive screening test to establish hypoxaemia [6]. A tcSO_2_ ≤ 97% has been shown to detect diagnostic PaO_2_ levels below 70 mmHg with 100% sensitivity and 46% specificity [17]. Other authors have, however, proposed that pulse oximetry is not sensitive to detect early stages of HPS that are more amenable to treatment and suggest screening by hyperaemic arterialised capillary blood gas analysis instead [18].

HPS is associated with high morbidity and mortality, especially after the onset of hypoxaemia with a baseline pO_2_ equal to or below 50 mmHg [19]. Currently, there are no medical therapies available, apart from liver transplantation (LT). LT has been reported to provide substantial improvement, with a postoperative normalization of alveolar gas exchange, as seen in our case, but carries a significant risk, which increases when there is an underlying metabolic condition affecting multiple vital organs, including the brain and heart. The decision to proceed with LT in children with underlying metabolic conditions requires careful consideration, balancing the risk of major surgery for affected children and for a potential living related donor against the expected improvement in quality of life and survival. In our case, the ongoing oxygen requirement and chronic hypoxaemia presented a significant risk of imminent deterioration. The previous stability and good quality of life of our patient influenced the decision in favor of transplantation.

LT can potentially ameliorate the clinical course of a generalized peroxisomal disorder. Van Maldegram reported a significant biochemical and symptomatic improvement in a 6‐month‐old child with mild ZSD who underwent orthotopic LT [20]. Menon et al. [21] also recently reported a successful LT in an 11‐year‐old with decompensated cirrhosis and bilateral proptosis, which resolved in the postoperative period. Demaret et al. [22] reported a series of three patients with mild ZSD who underwent LT and were followed up for 17 years. One of them died, while the other two displayed significant biochemical and neurodevelopmental improvement. They suggest that in mild ZSD, when LT is performed early in the course of the disease, it might enable a partial metabolic remission leading to long‐term clinical improvement.

We have seen a clear improvement in biomarkers of the disease after transplantation but apparent progression of retinopathy and neurological function in our patient. More data from other transplanted patients is required to determine whether and to which extent extrahepatic manifestations improve in the post‐transplant period.

In conclusion, HPS was an unexpected complication of apparently mild liver disease in our patient with intermediate ZSD. The patient had increased transaminases, gamma GT, and bilirubin, but sonographic features suggested only mild portal hypertension. The hepatic venous pressure gradient was, however, clearly increased, and the explanted liver showed advanced liver cirrhosis as a likely cause of HPS. It remains speculative whether there was any additional contribution to the development of HPS from toxic systemic effects or from intrinsic endothelial disease caused by peroxisomal dysfunction. The life‐threatening HPS could be successfully treated with a LT and enabled our patient to recover and remain in good health.

Awareness of HPS may be limited among pediatricians that are not specializing in liver disease. Because this complication can only be treated within a limited window of opportunity, screening for HPS in children with chronic liver disease should be considered during their follow‐up. Preliminary evidence suggests some effectiveness of LT in preventing progression of neurological disease in mild or intermediate ZSD.

## Author Contributions

Data collection, initial manuscript draft, final proofreading: Riya Mary Tharakan. Patient treatment, data collection, writing of manuscript, final proofreading: Sanjay Rajwal and Bernd C. Schwahn.

## Ethics Statement

All procedures followed were in accordance with the ethical standards of the responsible committee on human experimentation (institutional and national) and with the Helsinki Declaration of 1975, as revised in 2000 (5).

## Consent

Informed consent was obtained from the patient's legal guardians for being included in the study.

## Conflicts of Interest

The authors declare no conflicts of interest.

## Data Availability

Data supporting the results reported in the article can be requested from the authors upon reasonable request.
